# Dosimetric delivery validation of dynamically collimated pencil beam scanning proton therapy

**DOI:** 10.1088/1361-6560/acb6cd

**Published:** 2023-02-20

**Authors:** Nicholas P Nelson, Wesley S Culberson, Daniel E Hyer, Theodore J Geoghegan, Kaustubh A Patwardhan, Blake R Smith, Ryan T Flynn, Jen Yu, Alonso N Gutiérrez, Patrick M Hill

**Affiliations:** 1 Department of Medical Physics, School of Medicine and Public Health, University of Wisconsin—Madison, 1111 Highland Avenue, Madison, WI, 53705, United States of America; 2 Department of Radiation Oncology, University of Iowa Hospitals and Clinics, 200 Hawkins Drive, Iowa City, IA, 52242, United States of America; 3 Department of Radiation Oncology, Miami Cancer Institute, Baptist Health South Florida, 8900 N. Kendall Drive, Miami, FL, 33176, United States of America; 4 Department of Human Oncology, School of Medicine and Public Health, University of Wisconsin—Madison, 600 Highland Avenue, Madison, WI, 53792, United States of America

**Keywords:** proton therapy, collimation, Monte Carlo, dosimetry

## Abstract

*Objective*. Pencil beam scanning (PBS) proton therapy target dose conformity can be improved with energy layer-specific collimation. One such collimator is the dynamic collimation system (DCS), which consists of four nickel trimmer blades that intercept the scanning beam as it approaches the lateral extent of the target. While the dosimetric benefits of the DCS have been demonstrated through computational treatment planning studies, there has yet to be experimental verification of these benefits for composite multi-energy layer fields. The objective of this work is to dosimetrically characterize and experimentally validate the delivery of dynamically collimated proton therapy with the DCS equipped to a clinical PBS system. *Approach*. Optimized single field, uniform dose treatment plans for 3 × 3 × 3 cm^3^ target volumes were generated using Monte Carlo dose calculations with depths ranging from 5 to 15 cm, trimmer-to-surface distances ranging from 5 to 18.15 cm, with and without a 4 cm thick polyethylene range shifter. Treatment plans were then delivered to a water phantom using a prototype DCS and an IBA dedicated nozzle system and measured with a Zebra multilayer ionization chamber, a MatriXX PT ionization chamber array, and Gafchromic™ EBT3 film. *Main results*. For measurements made within the SOBPs, average 2D gamma pass rates exceeded 98.5% for the MatriXX PT and 96.5% for film at the 2%/2 mm criterion across all measured uncollimated and collimated plans, respectively. For verification of the penumbra width reduction with collimation, film agreed with Monte Carlo with differences within 0.3 mm on average compared to 0.9 mm for the MatriXX PT. *Significance*. We have experimentally verified the delivery of DCS-collimated fields using a clinical PBS system and commonly available dosimeters and have also identified potential weaknesses for dosimeters subject to steep dose gradients.

## 1. Introduction

Pencil beam scanning proton therapy (PBS-PT) is an increasingly common form of radiotherapy that utilizes a scanned beam of protons in successive energy layers to deliver a complex radiation dose distribution (Lomax 1999, Tian *et al* 2018). Target dose conformity in PBS-PT is dominated by the spot size, which is dictated by the standard deviation of the measured Gaussian fluence distribution in air at isocenter (${\sigma }_{{\mathrm{a}}{\mathrm{i}}{\mathrm{r}}}$) (Moignier *et al* 2016a, Grewal *et al* 2021, Nelson *et al* 2021a). Clinical spot sizes can be as small as 3 mm and greater than 20 mm at isocenter. Spot sizes depend on many factors such as the nozzle design, the energy selection system upstream, the requested beam energy, the beam divergence, and air gap between the treatment nozzle and patient surface (Gillin *et al* 2010, Titt *et al* 2010, Kralik *et al* 2015, Langner *et al* 2017, Smith 2019, Rahman *et al* 2020, Nelson *et al* 2021a). For example, Wang *et al* found that a spot size of less than 4.3 mm for PBS-PT was required to produce a superior treatment to state-of-the-art photon modalities for the treatment of intracranial lesions (Wang *et al* 2014). Widesott *et al* found that ${\sigma }_{{\mathrm{a}}{\mathrm{i}}{\mathrm{r}}}$ values must be less than 3 mm, 4 mm and 6 mm, to maintain clinical OAR doses relative to advanced photon therapy techniques for prostate, head and neck, and malignant mesothelioma cases, respectively. Many vendors have employed external collimation devices to reduce the effective spot size at the patient surface. Collimators used in PBS-PT include fixed per-field apertures (Moteabbed *et al* 2016, Winterhalter *et al* 2018, Maes *et al* 2019), multileaf collimators adapted from x-ray modalities, (Bues *et al* 2005, Daartz *et al* 2009, Daartz and Maughan 2014) and other novel collimation devices (Hyer *et al* 2014a, Kang and Pang 2020, Vilches-Freixas *et al* 2020, Chiang *et al* 2021, Grewal *et al* 2021). These devices have greatest impact on the treatment of shallow tumors, which require lower beam energies with larger spot sizes and range shifters that introduce considerable beam-broadening scatter.

A dynamic collimation system (DCS) is currently under development to reduce the lateral penumbra in PBS-PT to enable dynamically collimated proton therapy (DC-PT) (Hyer *et al* 2014a, Geoghegan *et al* 2020, Smith *et al* 2020). The DCS consists of two pairs of nickel trimmer blades (four total), an optional 4 cm thick polyethylene range shifter (WET = 4.1 cm^5^) to treat shallow (<4 cm) targets, and is mounted to a telescoping accessory nozzle that can produce various trimmer-to-surface distances (TSDs) ranging from 5 to 18.15 cm to accompany a wide array of patient and setup geometries. Each trimmer has a range of motion that provides up to 7.5 cm between the central axis of the beam coordinate system and the medial edge of the trimmer, resulting in a maximum field of view of 15 × 15 cm^2^ at isocenter. Polyethylene was selected as the range shifter material due to its minimal scattering power and thus, preservation of the in-air spot size and resulting dose conformity (Shen *et al* 2015). Nickel was selected for the trimmers based off previous results of Smith (2019), who showed that the secondary neutron ambient dose equivalence for PBS with the DCS was less than reported for uniform scanning with static brass apertures (Islam *et al* 2017, Smith *et al* 2019a).

Preliminary dosimetric and mechanical validation steps have been taken with the DCS mounted to the IBA dedicated nozzle (DN) system located at the Miami Cancer Institute (MCI), including the development of a Monte Carlo model and a central axis alignment device for trimmer positioning (Geoghegan *et al* 2022, Nelson *et al* 2021a). While the dosimetric benefits of DC-PT have been extensively evaluated through treatment planning studies, (Hyer *et al* 2014b, Smith *et al* 2016, Moignier *et al* 2016a, Moignier *et al* 2016b, 2016c) direct measurements of dynamically collimated proton dose distributions have yet to be performed. Therefore, the purpose of this work is to experimentally validate volumetric treatment plans with the DCS mounted to the IBA DN system using multiple detectors. This work will also provide an insight into detector performance for benchmarking the sharp dose gradients produced by the DCS.

## 2. Materials and methods

### 2.1. Treatment planning and dose optimization

The open-source Dynamic Collimation Monte Carlo package (Nelson *et al* 2021a), or DCMC, was used to commission TOPAS-based Monte Carlo models of the DCS and IBA DN nozzle to create and optimize three-dimensional uncollimated and collimated treatments for 3 × 3 × 3 cm^3^ cubic planning target volumes (PTVs). While this model has been previously validated against measurements of individual collimated beamlets, it has yet to be validated against composite treatment fields. Each PTV was placed at various depths to parametrize the penumbra sharpening as a function of depth in water. The impact of the trimmer-to-surface distance was also investigated for range shifted (RS) and non-range shifted (NRS) treatments. A summary of the target volumes and beam parameters investigated is in table 1.

**Table 1.** Summary of relevant plan parameters for 3 × 3 × 3 cm^3^ target volumes used to investigate penumbra sharpening as a function of the target’s center depth. Plan names are listed using a C*X*-D*Y*-*Z* nomenclature where *X* is the side length of the cube in cm, *Y* is the depth of the cube center in cm, and *Z* specifies if the plan utilized range shifted (RS) or non-range shifted (NRS) beamlets.

Cube size (cm^3^)	Depth (cm)	Range shifter present? (Y/N)	Number of energy layers	Energy range (MeV)	Plan name
	5	Y	11^a^	96.8–120.9	C3-D5-RS
3 × 3 × 3	6	N	12	71.4–100.6	C3-D6-NRS
	10	N	12	104.3–127.0	C3-D10-NRS
	15	N	12	136.2–155.7	C3-D15-NRS

^a^ Proximal layer dropped due to minimum MU constraints.

For each PTV, candidate beam spots were placed within a 2.5 mm expansion of PTV in the lateral and depth dimensions to ensure a uniform dose within the PTV. A 3 mm lateral in-plane spot spacing was utilized with a ∼3 mm energy layer spacing that resulted in 12 planned energy layers for all PTVs. For the collimated plans, the medial face of each trimmer was positioned to collimate the outer-most beamlets and trimmers were moved in between each energy layer to match the divergent field edge to the cubic target volume.

TOPAS (Perl *et al* 2012) (Version 3.8.p1) was used to simulate each candidate beamlet incident on a 40 × 40 × 40 cm^3^ water phantom and the dose to water was scored in Gy/proton on a 3D voxelized dose grid with a 1 mm isotropic resolution. Each plan consisted of 2028 candidate beamlets that were simulated using the computing resources and assistance of the UW-Madison Center for High Throughput Computing. Each simulation consisted of 1e6 histories per beamlet and utilized the default TOPAS physics modules: *g4em-standard_opt4, g4h-phy_QGSP_BIC_HP, g4decay, g4ion-binarycascade, g4h-elastic_HP, and g4stopping*.

Subsequent beamlet weight optimizations were performed using an in-house MATLAB-based linear least squares optimizer developed by Flynn *et al* (2007), (Flynn 2007) where the number of protons was optimized to provide a uniform dose to the PTV. Following the optimization process, the number of protons was converted to MU using a previously-established Monte Carlo model conversion factor that describes the number of protons per MU required to reproduce the IAEA TRS-398 reference dose measurement (Nelson *et al* 2021a, IAEA. IAEA TRS 398 2000). A uniform dose of 5 Gy was prescribed to the PTV to (1) minimize the amount of beamlets dropped due to minimum MU constraints during experimental validation while, (2) ensuring that a majority of the dose was within the dose calibration window of the EBT3 film that was later used to benchmark these dose distributions. The proximal energy layer for the range shifted case was removed following weight optimization due to the minimum MU constraint of 0.05 MU at the Miami Cancer Institute. Optimizations were performed to achieve identical 90% target prescription dose coverage between the uncollimated and collimated plans.

### 2.2. Treatment delivery

Following the treatment planning and optimization, spot positions, energies, and the optimized weights were used to write PBS layer definition (PLD) files that allow for delivery of scanned fields from the accelerator console in a physics/service mode. The PLD contains details of the energy required for each layer and, for each layer, the number of MUs and position for each spot. Because the optimized beamlet weights are in units of number of initially simulated protons, these weights were converted to MUs using an energy-dependent TRS-398 derived Monte Carlo conversion factor

\begin{eqnarray*}\displaystyle \frac{{\mathrm{p}}{\mathrm{r}}{\mathrm{o}}{\mathrm{t}}{\mathrm{o}}{\mathrm{n}}{\mathrm{s}}}{{\mathrm{M}}{\mathrm{U}}}=\displaystyle \frac{{D}_{{\mathrm{M}}{\mathrm{e}}{\mathrm{a}}{\mathrm{s}},{\mathrm{ref}}.}\left(\displaystyle \frac{{\mathrm{G}}{\mathrm{y}}}{{\mathrm{M}}{\mathrm{U}}}\right)}{{D}_{{\mathrm{M}}{\mathrm{C}},{\mathrm{ref}}.}\left(\displaystyle \frac{{\mathrm{G}}{\mathrm{y}}}{{\mathrm{p}}{\mathrm{r}}{\mathrm{o}}{\mathrm{t}}{\mathrm{o}}{\mathrm{n}}}\right)},\end{eqnarray*}

where ${D}_{{\mathrm{M}}{\mathrm{e}}{\mathrm{a}}{\mathrm{s}},\,{\mathrm{r}}{\mathrm{e}}{\mathrm{f}}.}$ and ${D}_{{\mathrm{M}}{\mathrm{C}},\,{\mathrm{r}}{\mathrm{e}}{\mathrm{f}}.}$ are the measured and simulated doses under TRS-398 reference conditions, respectively.

Prior to irradiation, the DCS trimmers were aligned to isocenter using the on-board x-ray imager to ensure accurate trimmer positioning. The trimmers were then moved to the first set of intended positions and verified with subsequent x-ray images. Trimmers were then moved in between energy layers using the ‘pause after layer’ function during the spot scanning delivery. While this did increase the overall measurement time, pausing between layers was necessary to move the trimmers because the DCS control system and IBA scanning controller system were operated independently. In the future, deliveries that require more than one trimmer position in an energy layer will require integration of the DCS control system with the IBA scanning controller, allowing for trimmer positions to be set prior to delivery of a given spot.

### 2.3. Experimental dosimetry

#### 2.3.1. Spread-out Bragg peak (SOBP) verification

Spread out Bragg peak (SOBP) profiles were measured for the NRS plans (6, 10 and 15 cm depths) using the Zebra multilayer ionization chamber (MLIC, IBA Dosimetry, Germany) to verify the SOBP range (depth of distal 90%), width, and uniformity of the evaluate plans agreed with the simulated SOBP distributions. The Zebra MLIC consists of 180 parallel plate ionization chambers (2.5 cm diameter) with an effective measurement resolution of 2 mm along the beam axis, and has been shown to be a quick and effective device for measuring proton range and SOBP width for both pristine beamlets and composite fields (Dhanesar *et al* 2013, Bäumer *et al* 2015). All collimated irradiations were performed using a 5 cm TSD.

#### 2.3.2. Lateral profile measurements

Lateral profiles of the cube dose distributions were measured using Gafchromic^TM^ EBT3 film and a MatriXX PT ionization chamber array (referred to as ‘MatriXX’ onward) in the DigiPhant PT (IBA Dosimetry, Germany) scanning water tank. Planar 2D measurements were performed and the center of the SOBP for each of the NRS plans investigated while EBT3 film was used to measure the range shifted plan. Additional near-surface measurement was taken with the MatriXX and film at a depth of 4.1 cm for the 15 cm deep cube to investigate a near-surface dose enhancement with collimation. The surface dose enhancement with collimation was further assessed for the other plans through lateral profile analysis of the Monte Carlo dose distributions at a depth of 5 mm by comparing the dose horns between the uncollimated and collimated dose distributions. Surface dose enhancement, mainly found near the target edges, is a result of the increased relative weights on collimated beamlets that also have an increased entrance dose on a per-beamlet basis from low-energy scattered protons originating from the trimmers.

**Figure 1.**
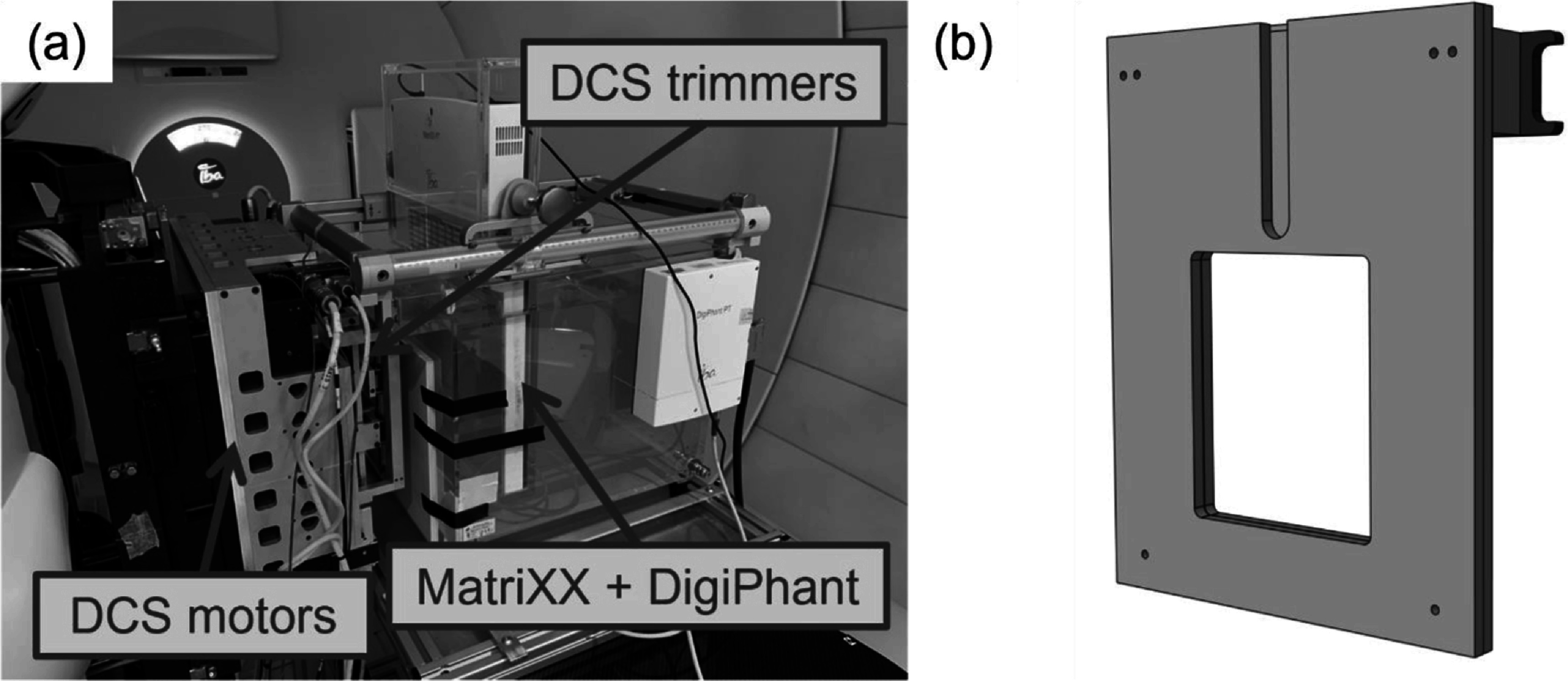
(a) Photograph of the MatriXX array and the DigiPhant water tank used for lateral profile measurements. (b) Rendering of DigiPhant-compatible EBT3 film holder.

The MatriXX, shown along with the DigiPhant water tank in figure 1(a), is a well characterized ion chamber array that utilizes 1020 vented parallel plate chambers with a 7.6 mm center-to-center spacing, each with an active volume of 32 mm^3^ (Arjomandy *et al* 2008, 2010, Lin *et al* 2015, Chan *et al* 2017). The effective measurement resolution with the MatriXX was increased by merging two datasets: one acquired with the MatriXX in its nominal position and another where the MatriXX was shifted in the horizontal and vertical directions by 3.8 mm, or half the distance between the ionization chambers. Merged datasets were then interpolated to a 1.5 mm dose grid for further analysis. Despite its extensive use for patient-specific quality assurance for passive scattering and PBS delivery, the MatriXX has yet to be used to benchmark extremely sharp dose gradients expected to be produced by the DCS (up to 20%/mm mid-SOBP).

Because of these gradients, high-resolution EBT3 film was utilized to corroborate MatriXX results. A custom polyethylene based EBT3 film holder was developed to mount film within the DigiPhant PT. The film holder, shown in figure 1(b), was designed such that the effective points of measurement between the film and MatriXX were identical and reproducible. The film holder was machined to conform to the specifications of 8′ × 10′ sheets of EBT3, and ultimately presents a 17 × 20 cm^2^ cross-sectional area of film for measurement directly in water. Following irradiation, films were read out using an Epson 10000XL flatbed scanner following irradiation and converted to dose to water using a 13-point 6 MV calibration curve created using a Varian TrueBeam Linear Accelerator at the University of Wisconsin Medical Radiation Research Center. Because of the known under response of EBT3 film in high proton LET environments (Smith *et al* 2019b), the use of EBT3 film in this work was limited to relative dosimetry and not used to verify absolute doses. Following irradiation, both the MatriXX and film dose distributions underwent a 2D rigid optimization consisting of rotations and translations was performed to align the measured profiles with the simulated profiles.

## 3. Results

### 3.1. Monte Carlo penumbra sharpening

**Figure 2.**
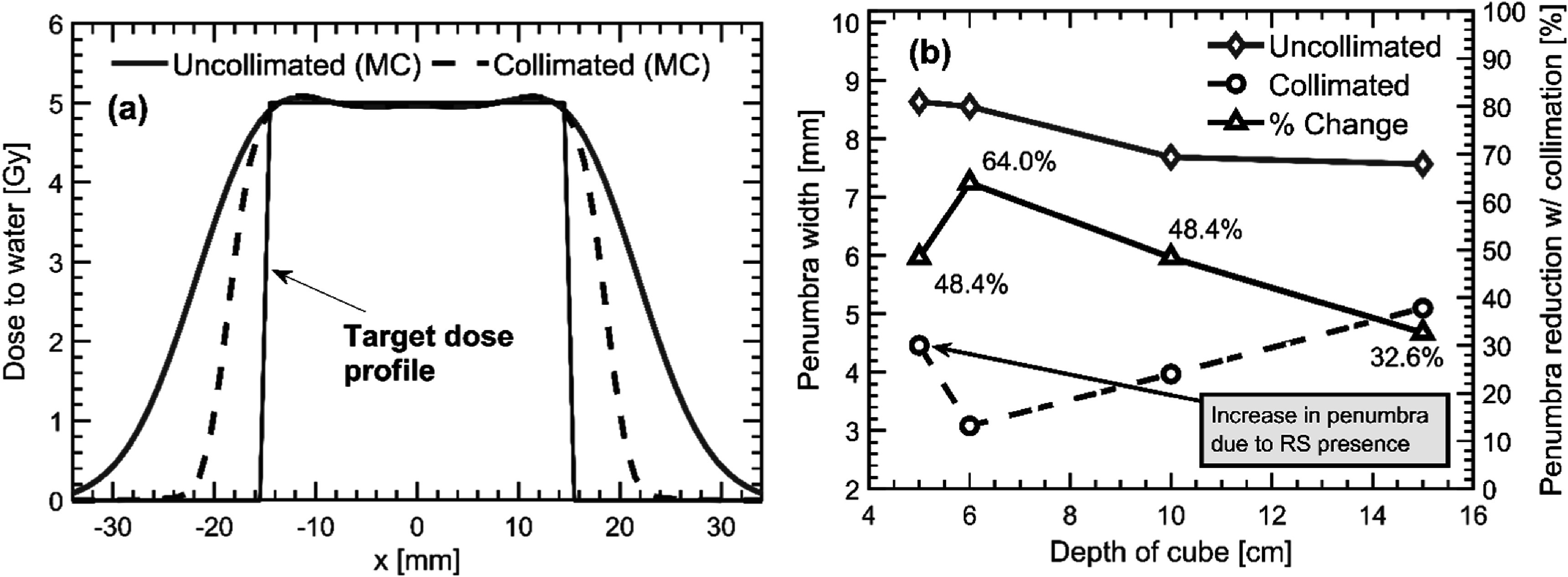
(a) Monte Carlo-simulated uncollimated (red, solid) and collimated (blue, dashed) lateral profiles at the center of the C3-D6-NRS target shown in black. (b) Simulated penumbra widths (uncollimated = red, solid; collimated = blue, dashed) are plotted as function of target depth on the left *y*-axis with percent change in penumbra width (black, solid) plotted on the right *y*-axis.

Following the beamlet weight optimization, penumbra widths (80%–20%) were tabulated as a function of target depth for the uncollimated and collimated plans. Figure 2 displays uncollimated and collimated lateral profiles for the C3-D6-NRS target plan (see table 2 for definition of plan name) and the Monte Carlo-simulated penumbra widths as a function of depth. For the NRS plans, penumbra reductions with the DCS ranged from 33% to 64%, or 2.5 to 5.5 mm, as the depth of the target decreases. For the RS plan at a depth of 5 cm, the penumbra was reduced by nearly 50%, or 4.2 mm.

**Table 2.** Summary of the simulated and measured penumbra widths (80%–20%) and penumbra width reductions with collimation for the plans and dosimeters investigated. The bottom row contains the average, absolute differences observed relative to the Monte Carlo values.

Cube Depth (cm)	Uncollimated (mm)			Collimated (mm)		
	MC	MatriXX	Film	MC	MatriXX	Film
6	8.4	8.6	7.7	3.0	4.4	2.7
10	7.6	8.0	7.2	3.9	5.0	3.8
15	7.5	7.9	7.2	5.0	5.9	5.1
15 (measured at 4.1 cm)	5.7	6.2	5.4	1.9	3.8	1.4
$\overline{\left|{\mathrm{MC}} \mbox{-} {\mathrm{Measured}}\right|}$(mm)	—	0.4	0.4	—	1.3	0.2

**Figure 3.**
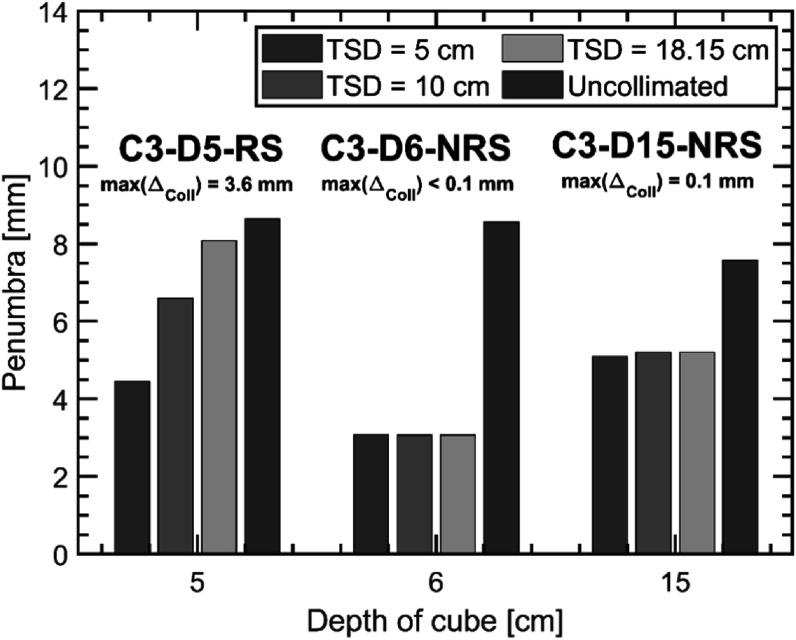
Bar chart of simulated penumbra widths for the C3-D5-RS, C3-D6-NRS, and C3-D15-NRS plans. Penumbra widths are shown for the uncollimated plans and the collimated plans under 5, 10, and 18.15 cm trimmer-to-surface distances (TSD).

Figure 3 displays the simulated penumbra widths for three of the treatments investigated in this work under various trimmer-to-surface distances (TSDs). The penumbra for the range shifted plan, C3-D5-RS, illustrates a large dependency on the TSD, where an increase in the penumbra width of 3.6 mm was seen between the 5 and 18.15 cm TSDs. A very weak to no correlation between the collimated penumbra widths and the TSD was observed for the NRS plans.

### 3.2. Experimental validation

#### 3.2.1. SOBPs

For the NRS plans shown in table 1, the depth of the distal 90% SOBP doses were verified to within 0.5 mm on average with a maximum difference of 0.7 mm and SOBP widths were within 1.5 mm on average with a max deviation of 1.7 mm.

#### 3.2.2. MatriXX and film profiles

**Figure 4.**
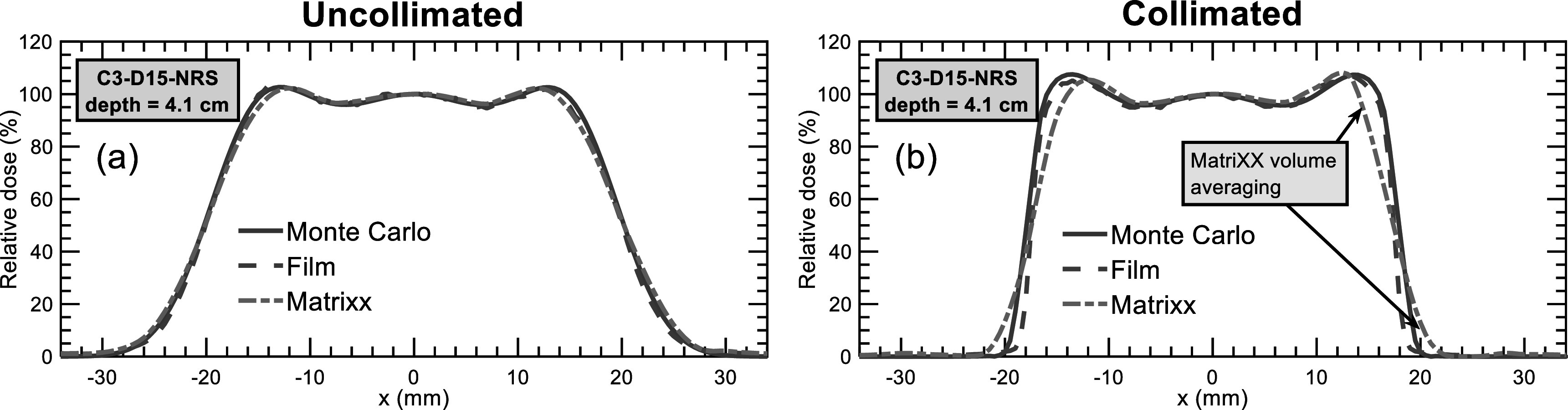
Monte Carlo, film, and MatriXX lateral profiles simulated and measured at a depth of 4.1 cm for the uncollimated (a) and collimated (b) 3 × 3 × 3 cm^3^ cube centered at a depth of 15 cm.

Figure 4 presents film and MatriXX lateral profile measurements for the uncollimated and collimated C3-D15-NRS plans at a depth of 4.1 cm. For the uncollimated case, excellent agreement is observed between simulation and measurements with 2D gamma (2%/2 mm/5% threshold) pass rates of 94.6% and 100% for comparisons with the MatriXX array and EBT3 film, respectively. When the MatriXX is used to measure the comparatively higher dose gradients for the collimated case, however, the pass rate is reduced to 83.7%, whereas film maintained a pass rate of 90.2%. MatriXX-measured penumbra widths (80%–20%) were 0.6 mm and 1.8 mm wider than Monte Carlo for the uncollimated and collimated dose distributions, respectively. For EBT3 film, these differences were reduced to 0.2 and 0.5 mm for the uncollimated and collimated plans, respectively.

**Figure 5.**
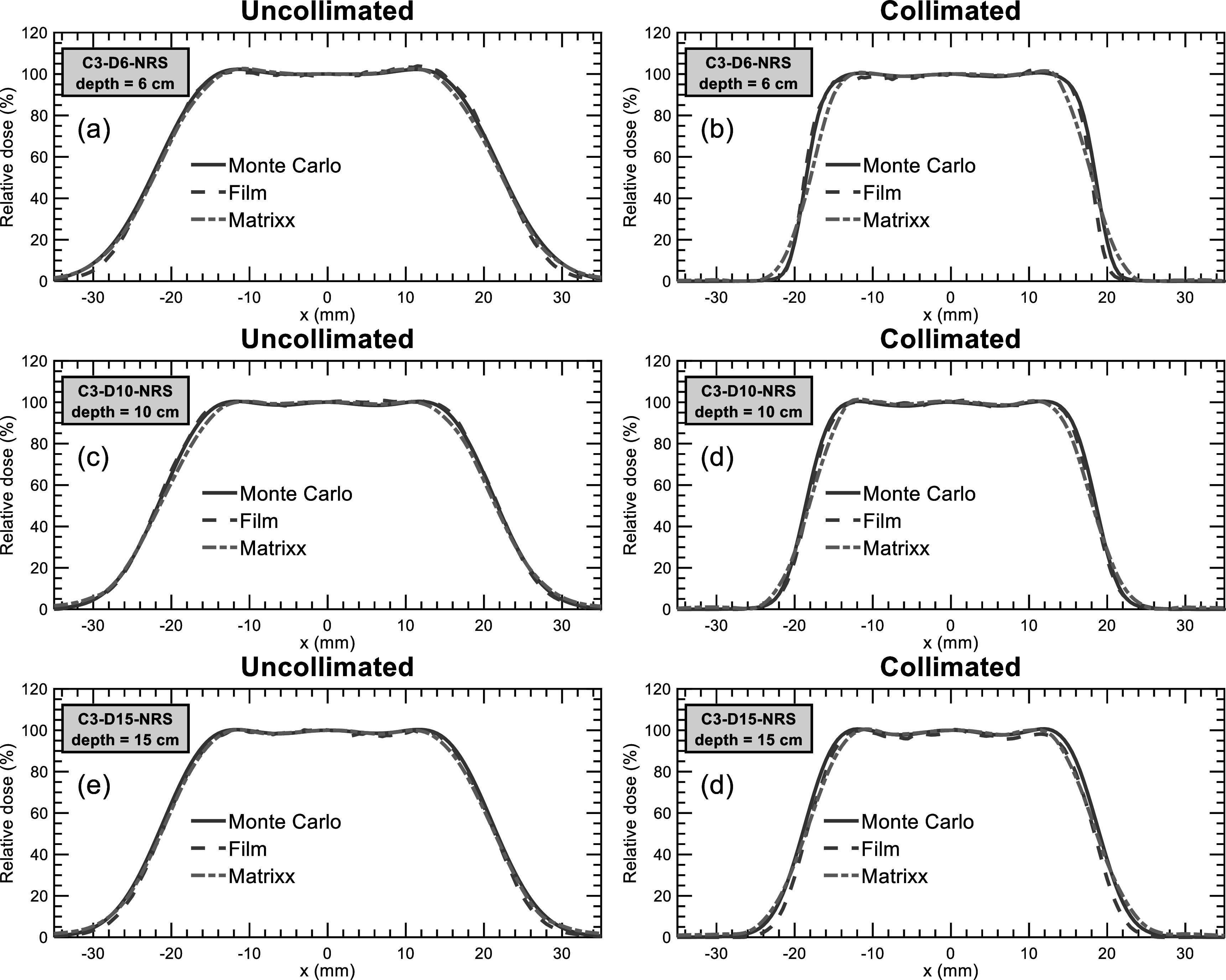
Monte Carlo, film, and MatriXX lateral profiles simulated and measured at the middle of the SOBPs for the C3-D6-NRS (top row), C3-D10-NRS (middle row), and C3-D15-NRS (bottom row) for uncollimated (left column) and collimated (right column) plan deliveries.

Figure 5 displays lateral dose profile results for measurements at the middle of the SOBPs for the uncollimated and collimated C3-D6-NRS, C3-D10-NRS, and C3-D15-NRS plans. Table 2 contains a comparison of the simulated and measured penumbra widths for the plans investigated. Uncollimated penumbra widths were determined to within 0.4 mm of Monte Carlo on average for both dosimeters while collimated penumbra widths were determined to within 1.3 and 0.2 mm for the MatriXX and EBT3 film, respectively. Penumbra reductions were measured to within 0.9 and 0.3 mm of Monte Carlo using the MatriXX and EBT3 film, respectively.

Relative 2D gamma pass rates are shown for the NRS plans and dosimeters investigated in table 3. Overall good agreement is observed, with average pass rates exceeding 96% across all planes of measurements, regardless of collimation. For the measurements performed proximal to the C3-D15-NRS SOBP, slightly better agreement was observed with EBT3 film, a result that can corroborated by the improved penumbra width agreement shown in table 2.

**Table 3.** Summary of relative 2D gamma pass rates between measurement (MatriXX and film) and simulation at a 2%/2 mm criterion with a 5% dose threshold applied on measured data points.

Cube Depth (cm)	Uncollimated		Collimated	
	MatriXX	Film	MatriXX	Film
6	100	97.0	99.0	100
10	100	100	99.3	100
15	100	93.3	98.0	91.0
15 (measured at 4.1 cm)	94.6	100	83.7	90.2

### 3.3. Surface dose enhancement

**Figure 6.**
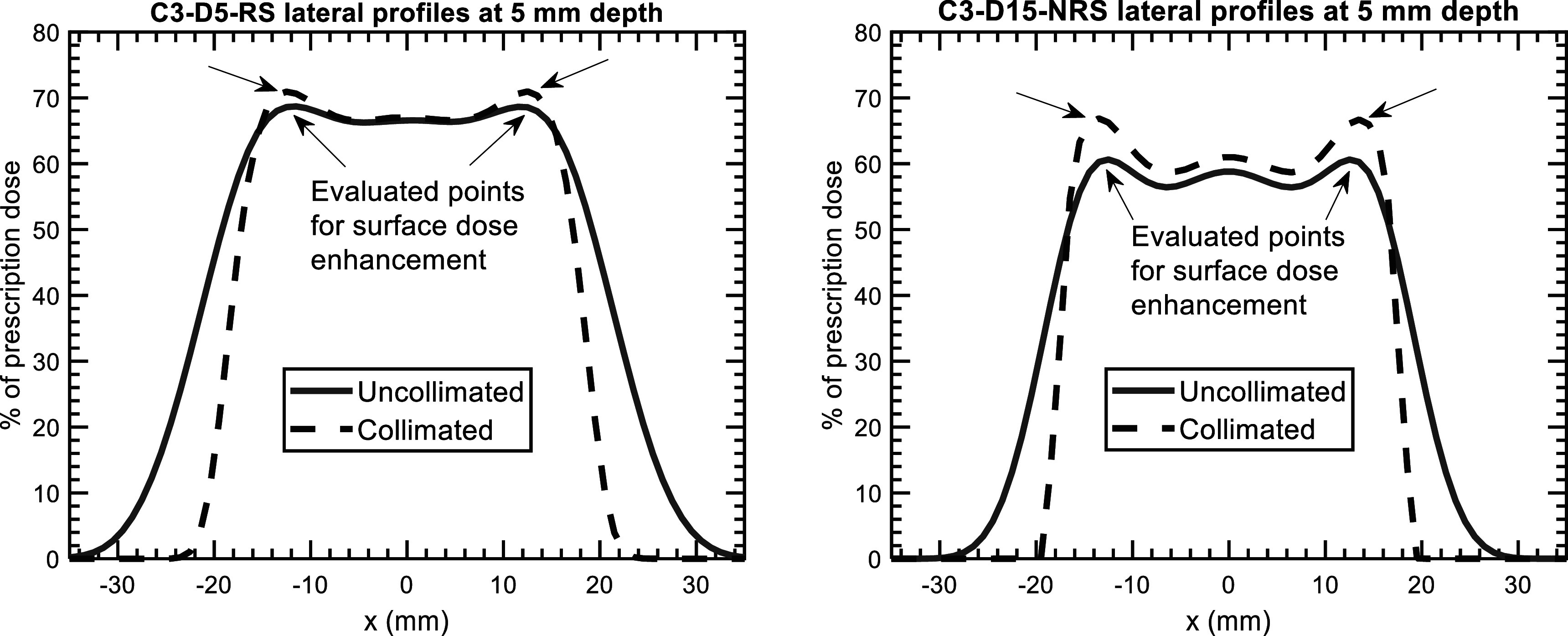
Simulated uncollimated and collimated lateral profiles at a depth of 5 mm for the C3-D5-RS and C3-D15-NRS plans. The arrows indicate the location of the evaluated dose points that were used to quantify the collimation-induced dose enhancement.

For the shallow measurements of the C3-D15-NRS plan, dose enhancements of 2.9 ± 1.6% and 1.3 ± 2.8% were observed using the MatriXX and EBT3 film. For the same plan, the Monte Carlo-simulated dose enhancement was 3.9 ± 0.4%, which is within the standard deviation of the MatriXX-measured values. To illustrate this surface dose enhancement effect, figure 6 displays lateral profiles for the C3-D5-RS and C3-D15-NRS plans at a depth of 5 mm. Table 4 summarizes the simulated dose enhancements at a depth of 5 mm for all plans investigated. In general, an increase in the dose enhancement was observed with increasing treatment depth.

**Table 4.** Monte Carlo-simulated dose enhancement at a depth of 5 mm for the plans investigated. Dose values are reported relative to the prescription dose and are the average of the max dose horns in each lateral dimension, for a total of four evaluated dose points per plan. Standard deviations are reported across the evaluated dose points. For the dose enhancement, collimated and uncollimated standard deviations were summed in quadrature.

Cube depth (cm)	Uncollimated dose (%)	Collimated dose (%)	Enhancement [Coll—uncoll] (%)
5 (RS)	68.6 ± 0.1	71.1 ± 0.1	2.5 ± 0.1
6	62.6 ± 0.1	64.6 ± 0.3	2.0 ± 0.3
10	59.7 ± 0.03	62.9 ± 0.5	3.2 ± 0.5
15	60.8 ± 0.2	67.6 ± 0.9	6.9 ± 0.9

## 4. Discussion

This work is the first to experimentally validate the penumbra reduction capabilities of the DCS equipped to a clinical proton therapy system using set of MU-optimized pencil beams. In the future, we aim to integrate the DCS control system with the beam scanning controller system to enable the delivery of treatment fields that require multiple trimmer positions in an energy layer rather than the single trimmer position per energy layer shown in this work. This work is a crucial step in the development of the DCS where the simulated penumbra sharpening capabilities of the DCS were compared to direct measurements of uncollimated and collimated treatment fields. This work has also served as an overall validation of the Monte Carlo planning and optimization techniques that were used to create the multi-spot treatment fields.

### 4.1. Monte Carlo simulations

For NRS plans, penumbra reductions ranged from 64% to 33% with increasing depth while the RS plan yielded penumbra reductions of around 50% at a depth of 5 cm in water. In general, uncollimated penumbral widths were below 9 mm and decreased with increasing depth, an effect that can be attributed to the reduced spot sizes and scatter cross sections for higher energy beams used to treat at deeper depths. For the collimated plans, an increase in the penumbra is evident with the inclusion of the range shifter (C3-D5-RS). This can be attributed to scattered protons originating from the range shifter that subsequently transmit through portions of the DCS trimmers. Once the range shifter is removed for the 6 cm depth and beyond, penumbra widths were all below 5.2 mm and found to linearly increase with depth at a rate of 0.23 mm per cm of water (*R* ^2^ = 1). The surface dose enhancement with the DCS was also observed to be no greater than 4.7% at a reference depth of 2 cm in water across all plans investigated.

Variation in TSD was found to impact the dose distribution only for fields that utilized the range shifter. This effect can be attributed to large-angle scattered protons originating from that range shifter that result in subsequent partial trimmer transmission, ultimately reducing penumbra widths on a per-beamlet basis, an effect that has been previously addressed in the context of analytical modeling of the DCS (Nelson *et al* 2022). These results also indicate a near-negligible influence of in-air scatter on the achievable penumbra width for DCS-collimated treatments over the range of TSDs evaluated.

### 4.2. Experimental validation

Experimental validation in this work consisted of measurements of the SOBP distributions and lateral profile measurements of the NRS plans. The Zebra MLIC was used to measure SOBPs, where the depths of the distal 90% dose point and SOBP widths were verified to within 0.5 and 1.5 mm, respectively. These results agree well with those previously reported by Dhanesar *et al* (2013), who benchmarked the performance of the MLIC against scanned ionization chamber measurements performed in water. Disagreements between measured and simulated SOBPs were observed in the plateau region and are a result of variation in the stopping power ratio between MLIC-equivalent material and water near the end of range (Nelson *et al* 2021b).

The MatriXX ionization chamber array and EBT3 film were used to measure lateral profiles at the middle of the SOBP for all plans and proximal to the SOBP of the C3-D15-NRS plan. Proximal measurements of this higher energy plan were performed to assess detector performance under the extremely steep dose gradients present at this depth, where the simulated penumbra width of the collimated plans was 1.9 mm, yielding a dose gradient of over 30% per mm. For lateral profile measurements at the middle of the SOBPs, overall excellent agreement was observed between Monte Carlo, MatriXX, and EBT3 film using 2D gamma analyses. For measurements performed proximal to the C3-D15 SOBP, EBT3 film agreed best with Monte Carlo in terms of 2D gamma analysis and penumbra width, whereas the MatriXX exhibited a significant amount of volume averaging as shown in figure 4(b) resulting in a lower gamma pass rate and wider penumbra width of greater than 2 mm. Considering clinical patient specific dosimetry is often performed within the SOBP using ionization chamber arrays, these findings support the use of the MatriXX for verification of collimated penumbrae within the SOBP for dose gradients up to 20% per mm, as seen in the C3-D6-NRS plan. For relative dose verification proximal to SOBPs, or dose gradients that exceed 20% per mm, the use of a high-resolution 2D dosimeter such as EBT3 film is recommended.

The surface dose enhancement with the DCS was also found to be small (<7%) at a depth of 5 mm for all plans and located at the lateral edges of the targets downstream from the trimmers. The surface dose enhancement with collimation arises from an increased surface dose on a per-beamlet basis that results from low-energy scattered protons penetrating to shallow depths in the phantom. This effect is also more pronounced due to the increased weighting placed on these beamlets that is required to achieve identical target coverage for the uncollimated case. Although the impact of scattered protons is relatively small for the cases investigated in this work, it is necessary to model in the context of a treatment planning system, where treatments that utilize larger, more complex sets of spot and trimmer positions will be calculated.

## 5. Conclusions

Predictions of collimated penumbra sharpening, previously limited to theoretical models and Monte Carlo simulation, have been confirmed by experimental measurements for a set of treatment plans that consider a range of scenarios relevant to expected clinical use of the DCS. The use of dynamic collimation can reduce penumbra by up to 60% as compared to deliveries with uncollimated spots, and the effect of the air gap on the achievable spot collimation is negligible if a range shifter is absent. Ion chamber array measurements are sufficient to characterize collimated gradient penumbrae in the SOBP for the set of plans measured, however higher resolution detectors such as film are required in proximal regions where gradients were more pronounced. Additional work is underway to determine the appropriateness of these detectors for more complicated treatment plans and spot patterns.

## Conflict of interest

DEH, RTF, and PMH are co-inventors on a patent that has been licensed to IBA.
